# A pilot study on traditional vs. digital self-administered questionnaire completion in the International Weight Control Registry

**DOI:** 10.1016/j.clnesp.2025.03.015

**Published:** 2025-03-13

**Authors:** Dimitrios Poulimeneas, Costas A. Anastasiou, Evgenia-Eleni Vlachogianni, Amalia Charokopou, Myrto Bitsa, Sai Krupa Das, Drew Sayer, Susan B. Roberts, James O. Hill, Mary Yannakoulia, the International Weight Control Registry Research Group

**Affiliations:** a Department of Nutrition and Dietetics, School of Health Sciences and Education, Harokopio University, Athens, Greece; b Jean Mayer, US Department of Agriculture, Human Nutrition Research Center on Aging at Tufts University, Boston, MA, USA; c The University of Alabama at Birmingham, School of Health Professions - Nutrition Sciences, 352000000, Birmingham, AL, USA; d Geisel School of Medicine, Dartmouth College, Hanover, NH 03755, USA

**Keywords:** Obesity, Web-based questionnaires, Weight loss, Weight loss maintenance, Weight regain

## Abstract

**Background & aim:**

Scarce reports on the characteristics of individuals choosing to enroll in obesity-related registries via digital or traditional methods exist. We examined whether characteristics of participants who enrolled in the International Weight Control Registry (IWCR) via digital surveys differed from those who enrolled with paper surveys.

**Methods:**

This manuscript describes the pilot phase of IWCR recruitment of eligible participants from one collaborating country. The IWCR includes questionnaires capturing biological, behavioral, environmental and psychosocial domains of weight management. All adult individuals with an intentional weight loss history, regardless of it being successful or not, are eligible to participate. Two invitations for participation were sent out. Firstly, potential participants were invited to complete the survey digitally; then, in the second invitation, potential volunteers were invited to complete the survey on paper.

**Results:**

A total of 69 persons with weight loss experiences completed this pilot survey (40 on-paper, 29 digitally). No differences were detected in the sex distribution and the level of education between groups (all p > 0.05). Fewer participants in the on-paper group reported to have experienced significant weight loss in their adult life (70.0 vs 100 %, p = 0.001). Maximum Body Mass Index (BMI) and initial weight loss (%) were similar in both groups, however the digital group reported significantly greater weight loss maintenance (86.5 vs 43.8 % of the initial weight loss, p = 0.008). More persons in the digital group reported to have achieved their current weight by linear weight loss (rapid or not) vs. through a series of weight loss cycles (p = 0.008).

**Conclusion:**

The different modes of engagement (digital vs. paper) may target different people. Traditional means of questionnaire completion were more appealing in individuals with less successful weight loss efforts. These preliminary results suggest that offering both online and paper versions of registry questionnaires may support data collection in a broader population demographic.

## 1. Introduction

Web-based questionnaires are being increasingly used instead of paper-based questionnaires in epidemiological surveys. Some advantages of web-based questionnaire include less time and cost to implement, as well as the ability to reach out to large audiences and track data completeness in near real-time [1]. In addition, surveys can be conducted completely remotely across a wide geographical area. On the other hand, compared to traditional approaches, web-based surveys have been reported to suffer from higher non-response rates (which may introduce selection bias), leading to concerns about data validity and reliability [1].

It is important to know if there are significant differences in characteristics of those who choose one method over the other. Literature suggests that persons with higher education are more likely to prefer digital means [2,3], while the role of sex and age is equivocal [3–5]. Data from nutritional epidemiology surveys, especially those aimed at individuals with obesity, are scarce. Therefore, it remains unknown which mode of questionnaire delivery may attract persons with weight loss experiences, of different weight status strata, and even more so those who have been less successful in losing weight or maintaining their weight loss. In preparing for efforts to engage Greeks in the International Weight Control Registry (IWCR), we first examined the characteristics of people engaging the IWCR through digital questionnaires vs those engaging with paper-based questionnaires. The study was implemented in the Greek cohort of the IWCR [6], an international partnership, with the aim to understand the diversity of factors associated with successful weight loss and weight loss maintenance in individuals and population groups. The IWCR’s over-arching goal is to identify factors that impact weight management.

## 2. Methods

### 2.1. The international weight control registry (IWCR)

The IWCR includes previously validated, or investigator-created (in areas where no validated instruments exist) questionnaires capturing biological, behavioral, environmental and psychosocial domains of weight management; all data are self-reported (a detailed summary of the IWCR questionnaires can be found else-where [6]). The IWCR can be accessed online, through its secure platform (www.internationalweightcontrolregistry.org). All adult individuals with an intentional weight loss history, regardless of it being successful or not, are eligible to participate. The IWCR has been granted approval by the institutional review board (IRB) of Tufts University (#13075). This manuscript describes the pilot phase of recruitment of eligible participants from Greece.

### 2.2. Sampling and questionnaire mode of delivery

Two invitations for participation in the IWCR were sent out through various means (i.e. word-of-mouth, university’s newsletter and social media, as well as contact with participants of another national weight maintenance registry). Firstly, potential participants were invited to complete the survey digitally; then, there was a second invitation to potential volunteers to complete the same survey on paper. In both modes of delivery, the questionnaires were the same. All questionnaires were translated in Greek using standardized procedures, and pilot-tested in 5 volunteers for clarity [6]. We sent out the link of the IWCR website to those that were chosen to fill in the web-based questionnaire, whereas we provided the on-paper questionnaire to those on the traditional approach. Informed consent was collected by all participants. All participants were given a deadline of one week to return the completed questionnaire.

### 2.3. Demographic & weight history measures

Basic demographics, such as sex, age (computed from date of birth and date of questionnaire completion), educational level (primary education, secondary education, graduate studies, post-graduate or doctoral studies), and marital status (single – never married, married, separated, divorced, widowed) were collected. Weight history questions included current weight and height, maximum weight, previous weight loss experiences, amount of weight loss achieved as an adult (if any), weight loss methods, weight loss achievement (through weight cycling, gradual or rapid weight loss). Body Mass Index was computed by dividing weight (in kg) with the squared height (in m). Initial weight loss was computed by subtracting the reported weight loss by the maximum weight, and was expressed as % maximum weight. Maintained weight loss was computed by subtracting the current weight from maximum weight, and was expressed as % of initial weight loss. All data were self-reported.

### 2.4. Statistics

Data are presented as means ± standard deviation of the mean for normally distributed variables and as medians and interquartile ranges (Q1, Q3) for skewed variables, or frequencies. Differences between continuous variables were examined with independent t-tests or Mann–Whitney U test (according to data distribution). Differences between categorical variables were examined with chi-square. A p-value ≤0.05 was considered significant.

## 3. Results

A total of 69 persons with weight loss experiences completed this pilot survey (40 on-paper, 29 digitally) (see Table 1). All questionnaires were returned within the given deadline, and the completeness of the questionnaires was 100 % in both groups. No differences were detected in the sex distribution and the level of education between groups (all p > 0.05). A trend for younger age in the digital group was noted (p = 0.08). Significantly more married individuals completed the questionnaire traditionally than digitally (42.5 vs. 10.3 %, p = 0.004).

With regards to weight history, fewer participants in the on-paper group reported to have experienced significant weight loss in their adult life (70.0 vs 100 %, p = 0.001). Maximum BMI and initial weight loss (%) were similar in both groups, however the digital group reported significantly greater weight loss maintenance (86.5 vs 43.8 % of the initial weight loss, p = 0.008). Less people in the traditional group were classified as maintainers of 5 % weight loss (p = 0.004). Last, significantly more persons in the digital group reported to have achieved their current weight by linear weight loss (rapid or not) vs. through a series of weight loss cycles, and the reverse was true for the persons in the on-paper group (p = 0.008).

## 4. Discussion

We identified some potentially significant differences between those who engaged with digital vs paper-based questionnaires. Data from this pilot investigation indicate that individuals less successful in their weight loss efforts are more likely to choose on-paper means for participating in the IWCR. Indeed, no weight loss regainer or person unsuccessful in losing weight chose the web-based version of the questionnaire, whereas 30 % of respondents of the on-paper group were unsuccessful in losing weight, and those who had lost weight had exhibited almost 2-fold weight regain compared to the digital group. Similarly, in the MedWeight study, a web-only weight control registry, the population of maintainers was much larger than that of regainers [7]. The difference in successful weight loss between the two groups (by method of choice in participating in the survey) may be explained by their motives for participation. Willingness and commitment in participating in a time-consuming effort is much higher for those reporting positive experiences [4]. Therefore, we speculate that successful weight losers devoted their personal time to fill in the survey primarily motivated by reporting their positive experience, whereas unsuccessful weight losers were mostly motivated by the obligation to hand-in the questionnaire to the study staff. This hypothesis is also strengthened by previous research that suggests that weight regainers have more external motives [8] for weight management.

In our study, sex, age and educational level of the participants did not differ significantly between groups, a finding also true for the respondents of a web-based or on-paper self-administered health questionnaire [5], as well as in a relevant survey in 3000 primary care psychology clients in the Netherlands [9]. On the other hand, the results of another study with the aim to investigate knowledge, attitudes and practices regarding infections indicate that more women, more well-educated persons and younger participants chose to deliver the questionnaires digitally than on-paper [3]. With regards to marriage, more married individuals chose the on-paper than the digital survey. This finding may be related to the previous reported evidence that marriage is more frequent in persons who have experienced weight regain [7].

Limitations of the present analysis stem from the observational design, the relatively small sample size, the self-selected population and the self-reported data. Although the relationship between sociodemographic characteristics and technology skills, as well as time and convenience reasons in choosing mode of completion cannot be overlooked, this is the first research item examining differences in demographics and weight history of respondents in a weight loss-related registry. At the same time, given the scarcity of the population in question, similar sampling and data collection techniques have been used by all weight control registries [10].

## 5. Conclusion

Our data provide some preliminary information for optimizing sampling techniques and minimizing selection bias in weight loss-related observational studies, especially in cohorts where stratified random sampling cannot be employed. The results, if confirmed by larger studies, suggest that offering both online and paper versions of registry questionnaires may support data collection in a broader population demographic.

## Figures and Tables

**Table 1:** Participants’ general characteristics, according to questionnaire completion mode (N = 69).

	On paper, n = 40	Digital, n = 29	p-value

*Demographics*			
Sex (% women)	72.5	69.0	0.749
Age (years)	30.4 (23.3, 48.3)	24.3 (22.1, 30.2)	0.080
Tertiary Education (%)	55.0	72.4	0.141
Married (%)	**42.5**	**10.3**	**0.004**
*Weight History*			
Maximum BMI (kg/m^2^)	27.6 ± 4.1	26.1 ± 4.4	0.772
Current BMI (kg/m^2^)	**25.9±4.2**	**22.6±2.8**	**0.001**
Had a great WL experience (%)	**70.0**	**100**	**0.001**
Greatest Initial WL (% max weight)	16.2 (13.3, 19.9)	13.3 (10.3, 21.4)	0.232
Maintained WL (% Initial WL)	**43.8 (0.0, 87.2)**	**86.6 (55.4, 99.1)**	**0.008**
Maintainer of 5 % WL (%)	**75.0**	**100**	**0.004**
Maintenance period			
<1 year	45.0	46.4	0.969
1–3 years	35.0	32.1	
>3 years	20.0	21.4	
Monitored by WL specialist (%)	35.0	17.9	0.121
WL Method			
Behavioral (%)	95.0	100	0.918
Pharmacotherapy (%)	5.0	0.0	0.506
Metabolic surgery (%)	0.0	0.0	–
Achievement of current weight			**0.008**
After a series of weight cycling (%)	**43.6**	**13.8**	
Through gradual WL (%)	**46.2**	**51.7**	
Through rapid WL (%)	**10.3**	**34.5**	

Bold font indicates significant differences.

BMI, Body Mass Index; WL, Weight Loss.
